# Psychological trauma and depression in recovered COVID-19 patients: a telecommunication based observational study

**DOI:** 10.47626/2237-6089-2021-0381

**Published:** 2023-05-02

**Authors:** Amit Srivastava, Renu Bala, Thokchom Priyobala Devi, Lily Anal

**Affiliations:** 1 Regional Research Institute Imphal Manipur India Regional Research Institute, Imphal, Manipur, India.

**Keywords:** COVID-19, depression, pandemic, posttraumatic stress disorder, psychological disorder

## Abstract

**Introduction:**

Coronavirus disease 2019 (COVID-19) is adversely affecting the mental health of patients infected with the virus and the psychological impact on recovered COVID-19 patients is unclear.

**Objectives:**

The study aimed to assess posttraumatic stress disorder (PTSD) and depression among COVID-19 patients after recovery from the disease.

**Methods:**

This cross-sectional study was conducted from November 9, 2020, to December 23, 2020. The study used a telemedicine model to enroll recovered COVID-19 patients from a database of patients provided by the health authorities. The National Stressful Events Survey PTSD Short Scale (NSESSS) for adults was used to assess PTSD symptoms and the Patient Health Questionnaire (PHQ-9) was used to assess depression.

**Results:**

The study enrolled 503 recovered COVID-19 patients with a mean age of 41.90 years. The majority were asymptomatic (64.6%), while 21.5% had had moderate to severe forms of the disease. Prevalence rates of PTSD symptoms and depression were 56.9 and 29% respectively. COVID-19 patients with severe forms of the disease were significantly more affected by PTSD symptoms (vs. mild, odds ratio [OR] = 18.7, 95%confidence interval [CI] 9.9-35.5) and depression (vs. mild, OR = 19.8, 95% CI 9.9-39.5). Similarly, patients who required oxygen or ventilator support reported significantly higher rates of PTSD symptoms (vs. managed at home, OR = 17.4, 95% CI 8.3-36.4) and depression (vs. managed at home, OR = 22.0, 95% CI 10.1-47.7).

**Conclusion:**

This study reports that recovered COVID-19 patients suffered from a significant amount of depression and experienced significant PTSD symptoms. It will help with addressing a major psychological concern among the recovered subjects.

## Introduction

Coronavirus disease 2019 (COVID-19), caused by a newly discovered coronavirus (severe acute respiratory syndrome coronavirus-2 [SARS-CoV-2]), was declared a pandemic by the World Health Organization (WHO).^1^ The COVID-19 pandemic reached unprecedented magnitude and India had recorded more than 10 million cases and 146,791 deaths as of late December 2020, when the present study was conducted.^2^ The pandemic had a significant effect on the psychological health of people in general and COVID-19 patients in particular, including fear, stress, anxiety, and depression that lead to social isolation and altered human relations.^3^ Studies of previous coronavirus (CoV) epidemics suggested that many patients with COVID-19 will manifest psychiatric symptoms and disorders.^4,5^ Fact-based and people-centric communication of pandemic-related information is indispensable to dispel fears and uncertainty among the population.^6^

The psychiatric illnesses may stem from psychosocial factors such as fear of infecting family members, lack of access to testing and medical care, physical distancing, home confinement, loneliness, economic hardships, and insecurity, etc.^4,7^ Studies on COVID-19 have shown a high prevalence of mental health problems among healthcare workers, the general population, students, and COVID-19 patients.^8-11^ Patients with severe COVID-19 are at risk of persistent psychiatric illness, since 20-40% of critically ill patients are known to manifest clinically significant symptoms, including anxiety, depression, and posttraumatic stress disorder (PTSD).^12-14^ While most of these mental health problems will fade out with time, symptoms of PTSD, a common mental disorder caused by major psychological trauma, may last for a long time and result in serious distress and disability.^15^

Varied prevalence of depression has been reported throughout the world in COVID-19 patients.^16,17^ A meta-analysis suggested evidence of depression, anxiety, and PTSD in the post-illness stage of a previous epidemics of SARS, but the current evidence regarding the prevalence of psychiatric disorders in CoV infected patients is scant and unclear.^18^ The COVID-19 pandemic has challenged the healthcare system to manage the crisis and provide the best possible healthcare facilities to patients.^19^ The present study aimed to assess the level of depression and PTSD in recovered COVID-19 patients.

## Methods

### Study design and setting

A cross-sectional study was conducted from November 9, 2020, to December 23, 2020, in Manipur State, India. The inclusion criteria for the study were that the participants should have recovered from COVID-19 (tested negative or completed an isolation period or discharged from a health care center after treatment), should have no previous history of PTSD or depression, should be over 18 years of age, of either gender, and should agree to participate in the study via telemedicine. Subjects with a previous history of PTSD or depression and those who did not agree to participate were excluded from the study.

### Study procedure

Participants were recruited for the study with a convenience sampling method. The investigators approached the district health authorities of Imphal and informed them about the study objectives and methods. The authorities were requested to provide the details of recovered COVID-19 patients in the city to enable selection of study subjects. The authorities shared the contact details of recovered patients, extracted from a database of COVID-19 patients in the city. A study representative contacted the recovered patients using telemedicine and apprised them of the objectives and purpose of the study. Subjects who were willing to give consent of their own free will were included in the study. Participants’ demographic information and the characteristics of their COVID-19 infection, including its manifestations and management were collected by the study representative using a pre-designed format. Interviews were conducted with recovered COVID-19 patients around 4 weeks after their recovery from the disease and the median interval between recovery and interview was 27 days. Each telephone interview with a recovered patient lasted around 15-20 minutes.

### Instruments

#### Severity of posttraumatic stress symptoms – Adult (National Stressful Events Survey PTSD Short Scale [NSESSS])

This is a validated nine-item measure that assesses the severity of PTSD symptoms in individuals following an extremely stressful event or experience. Each item asks the individual to rate the severity of his or her PTSD symptoms during the past 7 days on a five-point scale (0 = not at all; 1 = a little bit; 2 = moderately; 3 = quite a bit; and 4 = extremely). The total score can range from 0 to 36 with higher scores indicating greater severity of PTSD symptoms. If one or two items are left unanswered, a prorated score was calculated as per the scale instructions. The average total score reduces the overall score to a five-point scale, which categorizes the severity of PTSD symptoms as none (0); mild (1); moderate (2); severe (3); or extreme (4). Use of the average total score was found to be reliable, easy to use, and clinically useful to clinicians in the Diagnostic and Statistical Manual of Mental Disorders, 5th edition (DSM-5) field trials.^20^

#### Patient Health Questionnaire (PHQ-9)

This is a nine-item questionnaire used to evaluate depression that reflects the DSM-5 diagnostic criteria. Each item assesses the individual’s response over the previous 2 weeks with score options of 0 = not at all;, 1 = several days; 2 = more than half the days; and 3 = nearly every day. The total PHQ-9 score can range from 0 to 27 and, depending on the score obtained, it categorizes depression as minimal; mild; moderate; moderately severe; or severe.^21^

## Statistical analysis

The information gathered was entered into a spreadsheet and statistical tests were performed using Microsoft Excel software (Microsoft, Redmond, WA, USA). The descriptive analysis gave frequency and percentage of responses under each section. A chi-square test was performed to identify any significant differences in total PTSD and depression scores according to socio-demographic variables. The post-hoc test with Bonferroni’s correction identified groups that differed significantly from others. Binary logistic regression analysis was performed and odds ratios (OR) were calculated using StatCraft, version 2.0.3 (Bangalore, India), an online statistical software, to identify factors significantly associated with PTSD and depression faced by recovered COVID-19 patients. The significant level was set at p = 0.05.

## Ethics approval

The study was approved by the institutional ethical committee at the authors’ institute (reference no. 2-28/827). The study was conducted in accordance with the Declaration of Helsinki. The investigators from the institute requested data on recovered patients from the authorities and the request was approved by the competent state government authority. The contact details for recovered COVID-19 patients were thus duly transmitted to the research team and these data were only accessed by the research team and were maintained completely confidential.

## Results

### Participants

The study recruited 503 participants through telemedicine consultation with a mean (standard deviation [SD]) age of 41.90 (14.10) years. There was an almost equal participation by both genders with male participants accounting for 50.5% (n = 254) and female participants for 49.5% (n = 249). The majority of participants were married (n = 412; 81.9%) and lived in rural areas (n = 290; 57.7%). Furthermore, 39.8% (n = 200) had graduate qualifications and 30.2% (n = 152) were in a government or private job. Of the total sample of recovered COVID-19 patients, 31 (6.2%) had been pregnant when they got infected and 110 (21.9%) were already suffering from non-communicable disease (NCD) (Table S1, available as online-only supplementary material).

### Characteristics of COVID-19 in the participants

The majority of COVID-19 patients had been asymptomatic when infected (n = 325; 64.6%) and only 35.4% (n = 178) had developed disease symptoms. A moderate form of the disease developed in 9.9% (n = 50) of the patients, while the disease progressed to a severe stage in 11.5% (58) of the patients and 7.8% (n = 39) of COVID-19 patients required oxygen or ventilator support at the hospital. The majority of COVID-19 patients had taken allopathic prophylactic medicine (n = 287, 57.1%) before contracting the infection. Ayurveda, Yoga & Naturopathy, Unani, Siddha, and Homoeopathy (AYUSH) prophylactic medicines were taken by just six (1.2%) COVID-19 patients (Table S2, available as online-only supplementary material). The median time taken to recover from the disease was 11 days.

### PTSD and depression

The NSESSS score categorized the severity of PTSD symptoms in recovered COVID-19 patients as mild (n = 199, 39.6%), moderate (n = 65, 12.9%), and severe (n = 22, 4.4%), while none of the patients experienced an extreme form of PTSD (Table 1). Even after their recovery, some of the COVID-19 patients got emotionally upset on being reminded of the stressful experience (n = 129; 25.7%), and they had a very negative emotional state thereafter (n = 116; 23.1%). The participants (n = 121; 24.1%) tried to avoid thoughts, feelings, or physical sensations that reminded them of the stressful experience (Table S3). The chi-square analysis (Table 2) showed that PTSD symptoms were significantly more prevalent in married participants (p < 0.001) compared to unmarried individuals. There were significant differences in presence of PTSD symptoms among demographic groups selected by age (p < 0.001), education (p = 0.013), and presence of pre-existing medical conditions (p < 0.001). The post-hoc analysis (Table 3) was conducted after adjusting the p-value depending on the number of comparisons (Bonferroni adjustment). It showed that the observed frequency of PTSD symptoms was significantly higher among recovered COVID-19 patients above 60 years of age (p < 0.001) and among those who already suffered from an NCD before getting infected (p < 0.001). Similarly, the observed frequency of PTSD symptoms was significantly lower than expected among participants in the 18-30 years age group (p < 0.001) and those who were not suffering from an NCD before contracting the infection (p < 0.001).

**Table 1:** Prevalence of PTSD and depression in recovered COVID-19 patients

Depression severity	PHQ-9		PTSD severity	NSESSS	
	
	n	%		n	%
None or minimal	357	71.0	No	217	43.1
Mild	91	18.1	Mild	199	39.6
Moderate	31	6.1	Moderate	65	12.9
Moderately severe	18	3.6	Severe	22	4.4
Severe	6	1.2	Extreme	0	0.0

% = percentage; COVID-19 = coronavirus disease 2019; n = number; NSESSS = National Stressful Events Survey PTSD Short Scale; PHQ-9 = Patient Health Questionnaire; PTSD = posttraumatic stress disorder.

**Table 2:** Association between demographic variables and presence of PTSD and depression in recovered COVID-19 patients

Demographic variables	PTSD*						Depression^†^					

	Present		Absent				Present		Absent			
	n	%	n	%	χ^2^	p-value^‡^	n	%	n	%	χ^2^	p-value^‡^
Age (years)												
18-30	8	6.7	111	93.3	30.846	< 0.001	3	2.5	116	97.5	30.107	< 0.001
31-40	25	16.9	123	83.1			11	7.4	137	92.6		
41-50	13	15.5	71	84.5			10	11.9	74	88.1		
51-60	14	17.3	67	82.7			12	14.8	69	85.2		
Over 60	27	38.0	44	62.0			19	26.8	52	73.2		
Gender												
Male	41	16.1	213	83.9	0.478	0.489	29	11.4	225	88.6	0.123	0.726
Female	46	18.5	203	81.5			26	10.4	223	89.6		
Area												
Rural	46	15.9	244	84.1	5.687	0.058	25	8.6	265	91.4	12.377	0.002
Urban	34	23.0	114	77.0			27	18.2	121	81.8		
Semi-urban	7	10.8	58	89.2			3	4.6	62	95.4		
Marital status												
Married	83	20.1	329	79.9	12.926	< 0.001	55	13.3	357	86.7	13.639	< 0.001
Unmarried	4	4.4	87	95.6			0	0.0	91	100.0		
Education												
Postgraduate or higher	13	37.1	22	62.9	10.816	0.013	9	25.7	26	74.3	13.270	0.004
Graduate	29	14.5	171	85.5			15	7.5	185	92.5		
Up to higher secondary	30	16.5	152	83.5			17	9.3	165	90.7		
Up to primary	15	17.4	71	82.6			14	16.3	72	83.7		
Occupation												
Business/self employed	24	17.9	110	82.1	4.689	0.196	15	11.2	119	88.8	7.6281	0.054
Government/private job	25	16.4	127	83.6			16	10.5	136	89.5		
Housewife	31	21.4	114	78.6			22	15.2	123	84.8		
Unemployed	7	9.7	65	90.3			2	2.8	70	97.2		
Pre-existing medical condition												
NCD	36	4.6	74	9.5	27.308	< 0.001	26	3.3	84	10.7	25.970	< 0.001
No NCD	43	11.9	319	88.1			24	6.6	338	93.4		
Pregnancy	8	25.8	23	74.2			5	16.1	26	83.9		

% = percentage; COVID-19 = coronavirus disease 2019; n = number; NCD = non-communicable disease; PTSD = posttraumatic stress disorder.

χ^2^ = chi square test.

* PTSD based on National Stressful Events Survey PTSD Short Scale (NSESSS); ^†^ Depression based on Patient Health Questionnaire (PHQ-9); ^‡^ p < 0.05.

**Table 3:** Post-hoc analysis of NSESSS and PHQ-9 scores for presence of PTSD and depression in recovered COVID-19 patients

	Adjusted standardized residual value		z-value	p-value	Adjusted p-value (Bonferroni correction)

	Present	Absent			
PTSD					
Age (years)					
18-30	-3.490	3.490	-2.807	< 0.001	0.005
Over 60	4.984	-4.984			
Pre-existing disease					
NCD present	4.841	-4.841	-2.638	< 0.001	0.008
No NCD	-5.148	5.148		< 0.001	
Depression					
Age (years)					
18-30	-3.366	3.366	-2.807	< 0.001	0.005
Over 60	4.611	-4.611			
Area					
Urban	3.392	-3.392	-2.638	0.002	0.008
Education					
Postgraduate or higher	2.905	-2.905	-2.734	0.004	0.006
Pre-existing disease					
NCD present	4.830	-4.830	-2.638	< 0.001	0.008
No NCD	-4.957	4.957			

COVID-19 = coronavirus disease 2019; NCD = non-communicable diseases; NSESSS = National Stressful Events Survey PTSD Short Scale; PHQ-9 = Patient Health Questionnaire; PTSD = posttraumatic stress disorder.

Among the study participants, the severity of depression was assessed as mild (n = 91, 18.1%), moderate (n = 31, 6.2%), moderately severe (n = 18, 3.6%), and severe (n = 6, 1.2%) based on PHQ-9 scores (Table 1). After their recovery, the COVID-19 patients felt depressed, felt hopeless (n = 101; 20.1%), felt tired (n = 113; 22.5%), and experienced troubles with their sleep pattern (n = 84; 16.7%). The responses to the PHQ-9 questionnaire are given in Table S4, available as online-only supplementary material. The prevalence of depression (Table 2) was significantly higher among married participants (p < 0.001). There was a significant difference in presence of depression among demographic groups selected by age (p < 0.001), type of area (p = 0.002), education (p = 0.004), and pre-existing medical condition (p < 0.001). The post-hoc analysis (Table 3) showed that the observed frequency of depression was significantly higher among recovered COVID-19 patients over 60 years of age (p < 0.001), living in urban areas (p = 0.002), with postgraduate and higher education (p = 0.004), and those who already suffered from an NCD before getting infected (p < 0.001). Similarly, the observed frequency of depression was significantly lower than expected among participants of the 18-30 years age group (p < 0.001) and those who were not suffering from any NCD before contracting the infection (p < 0.001).

Logistic regression analysis (Table 4) indicated that participants who suffered from a severe form of the disease (vs. mild, OR = 18.7, p < 0.001; vs. moderate, OR = 9.4, p < 0.001), those who were symptomatic (vs. asymptomatic, OR = 2.6, p < 0.001), those who required oxygen or ventilator support (vs. managed at home, OR = 17.4, p < 0.001; vs. managed at home with medicines, OR = 4.7, p < 0.001) and those with a longer recovery time (OR = 2.2, p = 0.001) were significantly associated with presence of PTSD symptoms. Similarly, participants who suffered from a severe form of the disease (vs. mild, OR = 19.8, p < 0.001; vs. moderate, OR = 6.1, p < 0.001), those who required oxygen or ventilator support (vs. managed at home, OR = 22.0, p < 0.001), and those with a longer recovery time (OR = 2.9, p = 0.001) were significantly associated with presence of depression.

**Table 4:** Results of logistic regression analysis showing factors related to COVID-19 infection significantly associated with presence of PTSD and depression in recovered patients

Characteristics of COVID-19 in recovered patients	PTSD			Depression		

	OR	95%CI	p-value*	OR	95%CI	p-value*
Type of infection						
Severe vs. mild	18.7	9.9-35.5	< 0.001	19.8	9.9-39.5	< 0.001
Severe vs. moderate	9.4	3.8-23.1	< 0.001	6.1	2.4-15.9	< 0.001
Presentation						
Symptomatic vs. asymptomatic	2.6	1.6-4.1	< 0.001	3.7	2.1-6.7	< 0.001
Management						
Medicine + oxygen/ventilator vs. home	17.4	8.3-36.4	< 0.001	22.0	10.1-47.7	< 0.001
Medicine + oxygen/ventilator vs. home + medicine	4.7	2.0-11.0	< 0.001	4.4	1.9-10.5	0.001
Recovery time						
> Median vs. ≤ median	2.2	1.4-3.6	0.001	2.9	1.6-5.2	< 0.001

95%CI = 95%confidence interval; COVID-19 = coronavirus disease 2019; LCL = lower confidence limit; OR = odds ratio; PTSD = posttraumatic stress disorder; UCL = upper confidence limit.

* p < 0.05.

## Discussion

With the spread of the pandemic, the psychiatric implications of the disease are being widely acknowledged. COVID-19 patients, from fear of severe disease consequences, may experience anxiety, depression, PTSD, and other psychiatric complaints. The survivors of the pandemic are at risk of developing significant physical, cognitive, and psychological morbidities after recovery from COVID-19. The present study focused on the psychological impact of COVID-19, especially PTSD and depression, on patients who got infected with the virus but recovered after treatment.

Very few studies have been conducted across the globe and specifically in India to assess the prevalence of PTSD and depression in recovered COVID-19 patients. The National Mental Health Survey of India (NMHS) 2015-16 reported the overall prevalence of PTSD as 6.3% among the general population in Manipur state.^22^ The findings of the present study revealed that 56.9% of the recovered COVID-19 patients suffered from some form of PTSD, among whom 17.3% experienced a moderate to severe form of PTSD. A study with SARS survivors found a 47.8% prevalence of PTSD,^23^ while another study reported 66% PTSD prevalence among Ebola survivors.^15^ Studies of diagnosed COVID-19 patients have reported PTSD prevalence as 25-31%,^24-26^ and one systematic review found the prevalence of PTSD to be 32.2%.^18^ A very low prevalence of PTSD (3.8%) was reported in recovered COVID-19 subjects in a study from Iran.^27^ No significant difference in PTSD symptoms between female and male gender was found in the present study, similar to what was reported in a study from China,^28^ while few studies and a systematic review reported that the female sex was more susceptible to developing symptoms of PTSD.^18,29,30^ The older age group was more prone to develop PTSD symptoms as suggested by the findings of other studies among COVID-19 survivors.^26,27^

A 29% prevalence of depression among COVID-19 survivors was found in this study, with 10.9% of subjects experiencing a moderate to severe form of depression. The NMHS 2015-16 reported a 9.1% overall prevalence of depression among the general population in Manipur state.^22^ A high prevalence of depression was also reported during the SARS outbreak.^23,31^ Similarly, studies with COVID-19 survivors have reported prevalence of depression ranging from 23 to 38%.^26,32^ Most depression in this study was of mild severity, similar to the findings of another study.^33^ A study from China reported a very high prevalence of depression (60.2%) among COVID-19 patients,^16^ while, on the contrary, a study from Iran reported only 5% prevalence.^27^ The present study reported that patients in the older age group, particularly those over 60 years of age, were significantly more affected with depression, similar to another study,^32^ while a third study reported depression to be significant in COVID-19 patients in the younger age group.^27^ Studies reported significantly higher levels of depression among females,^27,32^ contrary to the present study, in which no significant difference in gender demographic groups was detected. The PTSD symptoms and depression in this study were significantly higher in patients who required oxygen or ventilator support compared to those managed at home. Similar findings were reported from Morocco, where depression was significantly related to the hospital stay.^32^

The number of asymptomatic COVID-19 patients is strikingly high (64.6%), which corroborates many other studies worldwide.^34,35^ Another finding that came to light in this study was about the use of prophylactic medicine from all streams of medical science taken by the participants. The majority of the participants (57.1%) took allopathic prophylactic medicine for COVID-19, while only 1.2% of the participants took AYUSH prophylactic medicine, as advised by the Ministry of AYUSH, Government of India (GOI).^36^ Claims have been made about the efficacy of AYUSH medicines as prophylactic against COVID-19 and as protecting the people from getting infected.^37,38^ However, researchers in India are still actively engaged in conducting clinical research to counter the pandemic. The clinical trials registered for COVID-19 with the Clinical Trials Registry of India (CTRI) have been categorized into modern medicine (n = 42), traditional medicine (n = 67), and miscellaneous (n = 13). The traditional medicine trials category majoritarily comprised Ayurveda (n = 45), followed by homeopathy (n = 14) and others (n = 8) from Yoga, Siddha, and Unani. Among the traditional medicine category, 31 trials were prophylactic and 36 were therapeutic, mostly conducted on asymptomatic or mild to moderate COVID-19 patients.^39^

Countries with minimal resources need to implement appropriate mental and physical health prevention measures. Strategies, such as a psychological helpline, may be adopted to facilitate citizens seeking mental health advice and can easily maintain communication for required timely assistance.^40^ Fear and uncertainty about the long-term adverse consequences after infection is contributing towards negative emotions among recovered COVID-19 patients. The fast-spreading pandemic is leading to higher psychological morbidity among recovered patients, who are also vulnerable to social discrimination. The findings of this study demonstrate significant evidence that may be beneficial for optimal clinical intervention needed by recovered subjects. Targeted epidemiological and clinical studies with varied demographic profiles need to be planned to understand the attributes and damaging effects of the pandemic in recovered COVID-19 subjects. Mental health support and psychological services should be tailored in light of the enforced restrictions and preventive methods.

Certain limitations to the present study shall be considered. First, this study was conducted on COVID-19 patients who had recovered from the infection in Imphal, a city in Manipur state, India, and, further, these subjects were recruited from a database of patients maintained by the health authorities, thus limiting the results to a certain socio-demographic and cultural profile. Assessment of a wider population of recovered COVID-19 patients must be done for an accurate picture of their psychological problems. Second, a convenience sampling method may bias the data and may not be representative of the population. Third, the data were collected over the telephone so there are possibilities of information bias and also selection bias due to network connectivity. Fourth, history of previous mental illness was not collected, limiting association of symptoms to COVID-19. Finally, being a cross-sectional study, follow-up of the subjects could not be undertaken. Studies are needed to determine the possible long-term adverse consequences for the psyche of recovered COVID-19 patients. Despite these limitations, this study presents an epidemiological analysis of COVID-19 cases that generates robust evidence on psychological problems faced by patients after recovery from COVID-19 and it is important that these findings need to be confirmed in a larger sample of patients.

### Implications

COVID-19 has infected a significant number of people globally and the psychological impact on recovered patients could also be a major concern. Mental illness may be compounded by the fear associated with a disease that leads to social isolation of the affected individuals. PTSD and depression have emerged as psychological morbidities of worrisome nature and the severity of COVID-19 is proportional to the severity of mental illness, as detailed in this study. The physical illness compounded by psychological morbidity may result in deterioration of the individual’s quality of life. This study has generated evidence that presents implications for formulating an effective strategy for screening of recovered COVID-19 patients who developed mental illness. Efforts should be made to provide easily accessible psychological counselling services for such individuals and proper healthcare facilities for those who require medical treatment.

The present study also highlighted the importance of generating further evidence on the long term psychological effects of COVID-19 and to further assess the correlation between the severity of COVID-19 and mental illness. Disseminating credible information about the probable psychological consequences of the disease to COVID-19 patients in particular and to the public in general should also be a priority for healthcare providers and public health authorities.

## Conclusion

This study has found that COVID-19 patients suffered from a significant amount of PTSD and depression after recovery and thus has helped by addressing major psychological concerns faced by them. The results obtained from this study could contribute to formulating effective strategies for comprehensive health care for recovered COVID-19 patients with specific emphasis on the psychological aspect. Considering the alarming impact of COVID-19 on the mental health of recovered patients, it may be suggested that their psychopathology should be assessed and their psychiatric conditions diagnosed and treated to reduce the disease burden. It is assumed that the outcomes of this study will work as a baseline for future studies in this context.
