# Supplementary material for: Psychological trauma and depression in recovered COVID-19 patients: a telecommunication based observational study
Source: Trends Psychiatry Psychother. 2023 May 2;45:e20210381. doi: 10.47626/2237-6089-2021-0381 (PMC10226770; doi:10.47626/2237-6089-2021-0381)
Supplement: Supplementary file 1 [file 2238-0019-trends-45-e20210381-suppl01.pdf]

http://doi.org/10.47626/2237-6089-2021-0381

Supplementary Table S1 - Socio-demographic characteristics of the recovered COVID-19 patients

**Demographic variable**	**n**	**%**
Mean age: 41.90		
Standard deviation: 14.10		
Age (years)		
18-30	119	23.7
31-40	148	29.4
41-50	84	16.7
51-60	81	16.1
Over 60	71	14.1
Gender		
Male	254	50.5
Female	249	49.5
Area		
Rural	290	57.7
Urban	148	29.4
Semi-urban	65	12.9
Marital status		
Married	412	81.9
Unmarried	91	18.1
Education		
Postgraduate	35	7.0
Graduate	200	39.8
Up to higher secondary	182	36.2
Up to primary	86	17.1
Occupation		
Business/self employed	134	26.6
Government/private job	152	30.2
Housewife/homemaker	145	28.8
Unemployed	72	14.3
Pre-existing medical condition		
NCD	110	21.9
No NCD	362	72.0
Pregnancy	31	6.2

% = percentage; COVID-19 = coronavirus disease 2019; n = number; NCD = non-communicable diseases.

http://doi.org/10.47626/2237-6089-2021-0381

Supplementary Table S2 - Characteristics of coronavirus disease 2019 (COVID-19) in the infected study participants

**COVID-19 characteristics**	**n**	**%**
Type of infection		
Mild	395	78.5
Moderate	50	9.9
Severe	58	11.5
Presentation		
Asymptomatic	325	64.6
Symptomatic	178	35.4
Management		
Home	397	78.9
Hospitalized + medicine	67	13.3
Hospitalized + medicine + oxygen/ventilator	39	7.8
Prophylactic medicine		
Allopathic	287	57.1
Ayurvedic	1	0.2
Homoeopathic	2	0.4
Local/folk	3	0.6
No medicine taken	210	41.8

% = percentage; n = number.

http://doi.org/10.47626/2237-6089-2021-0381

Supplementary Table S3 - Responses to severity of posttraumatic stress symptoms – Adult NSESSS in recovered COVID-19 patients

	Not at all		A little bit		Moderately		Quite a bit		Extremely	
	**n**	**%**	**n**	**%**	**n**	**%**	**n**	**%**	**n**	**%**
Having “flashbacks” (for example, you re-experienced parts of a stressful experience by seeing, hearing, smelling, or physically feeling parts of the experience)?	281	55.9	145	28.8	52	10.3	16	3.2	9	1.8
Feeling very emotionally upset when something reminded you of a stressful experience?	210	41.7	164	32.6	79	15.7	36	7.2	14	2.8
Trying to avoid thoughts, feelings, or physical sensations that reminded you of a stressful experience?	269	53.5	113	22.5	71	14.1	34	6.8	16	3.2
Thinking that a stressful event happened because you or someone else (who didn’t directly harm you) did something wrong or didn’t do everything possible to prevent it, or because of something about you?	227	45.1	155	30.8	81	16.1	24	4.8	16	3.2
Having a very negative emotional state (for example, you were experiencing lots of fear, anger, guilt, shame, or horror) after a stressful experience?	233	46.3	154	30.6	63	12.5	32	6.4	21	4.2
Losing interest in activities you used to enjoy before having a stressful experience?	334	66.4	78	15.5	47	9.3	30	6.0	14	2.8
Being “super alert”, on guard, or constantly on the lookout for danger?	60	11.9	144	28.6	111	22.1	110	21.9	78	15.5
Feeling jumpy or easily startled when you hear an unexpected noise?	323	64.2	24	4.8	2	0.4	0	0.0	0	0.0
Being extremely irritable or angry to the point where you yelled at other people, got into fights, or destroyed things?	439	87.3	50	9.9	12	2.4	1	0.2	0	0.0

% = percentage; COVID-19 = coronavirus disease 2019; n = number; NSESSS = National Stressful Events Survey PTSD Short Scale; PTSD = posttraumatic stress disorder.

http://doi.org/10.47626/2237-6089-2021-0381

Supplementary Table S4 - Responses to PHQ-9 questionnaire for assessment of depression in recovered COVID-19 patients

	Not at all		Several days		More than half the days		Nearly every day	
	n	%	n	%	n	%	n	%
1. Little interest or pleasure in doing things	341	67.8	95	18.9	54	10.7	13	2.6
2. Feeling down, depressed, or hopeless	250	49.7	152	30.2	77	15.3	24	4.8
3. Trouble falling or staying asleep, or sleeping too much	326	64.8	93	18.5	65	12.9	19	3.8
4. Feeling tired or having little energy	248	49.3	142	28.2	71	14.1	42	8.3
5. Poor appetite or overeating	326	64.8	110	21.9	48	9.5	19	3.8
6. Feeling bad about yourself – or that you are a failure or have let yourself or your family down	346	68.8	94	18.7	47	9.3	16	3.2
7. Trouble concentrating on things, such as reading the newspaper or watching television	437	86.9	55	10.9	9	1.8	2	0.4
8. Moving or speaking so slowly that other people could have noticed? Or the opposite – being so fidgety or restless that you have been moving around a lot more than usual	477	94.8	19	3.8	6	1.2	1	0.2
9. Thoughts that you would be better off dead or of hurting yourself in some way	483	96.0	11	2.2	6	1.2	3	0.6

% = percentage; COVID-19 = coronavirus disease 2019; n = number; PHQ-9 = Patient Health Questionnaire.
